# Endophytic Bacterial Exopolysaccharide and Ethylene Hyperactivate Flavonoid Synthesis in Leaves of *Fagus sylvatica* L. with False Heartwood

**DOI:** 10.3390/plants15162420

**Published:** 2026-08-07

**Authors:** Artur Likhanov, Svitlana Bilous, Iryna Smetanska, Maksym Kharkhota, Oleksandr Subin, Volodymyr Gryb, Vira Boroday, Liubov Zelena, Mariia Shevchuk, Andrii Bilous

**Affiliations:** 1 Botany, Dendrology and Forest Tree Breeding Department, National University of Life and Environmental Sciences of Ukraine, 15 Heroiv Oborony, 03041 Kyiv, Ukraine; 2 Institute for Evolutionary Ecology NAS of Ukraine, 37 Lebedeva Str., 03143 Kyiv, Ukraine; 3 Faculty of Horticulture and Food Science, Weihenstephan-Triesdorf University of Applied Sciences, Am Hofgarten 4, 85354 Freising, Germany; 4 Department of Production and Processing of Food, Weihenstephan-Triesdorf University of Applied Sciences, Am Hofgarten 4, 85354 Freising, Germany; iryna.smetanska@hswt.de; 5 D.K. Zabolotny Institute of Microbiology and Virology, National Academy of Sciences of Ukraine, 154 Zabolotnogo Str., 03143 Kyiv, Ukraine; m.kharkhota@imv.org.ua (M.K.); zelena@nas.gov.ua (L.Z.); 6 Ecobiotechnology and Biodiversity Department, National University of Life and Environmental Sciences of Ukraine, 15 Heroiv Oborony, 03041 Kyiv, Ukraineveraboro@gmail.com (V.B.); 7 Silviculture Department, National University of Life and Environmental Sciences of Ukraine, 15 Heroiv Oborony, 03041 Kyiv, Ukraine; 8 Institute of Agroecology and Environmental Management, National Academy of Agrarian Sciences of Ukraine, Metrologichna Str. 12, 03143 Kyiv, Ukraine; 9 Forestry Faculty, Weihenstephan-Triesdorf University of Applied Sciences, Am Hofgarten 4, 85354 Freising, Germany; 10 Department of Garden Design and Forestry, Sumy National Agrarian University, 160 Herasym Kondratiev Str., 40021 Sumy, Ukraine

**Keywords:** European beech, *Bacillus halotolerans*, elicitor, ethephon, endophytes, xylem, priming, stress

## Abstract

The presence of false heartwood in the stems of *Fagus sylvatica* L. significantly reduces timber quality; to date, its physiological triggers remain understudied. Research on beech stands in the forests of the Bukovinian Pre-Carpathians (Ukraine) was aimed at identifying functional links between leaf structure, synthesis of polyphenols, microbial elicitors, and false heartwood formation. Using morphometric analysis, epifluorescence microscopy, and spectrophotometry, trees with false heartwood (FH morphotype) were compared with those without it, the healthy control (HC morphotype). Sequencing of the 16S rRNA gene identified the endophytic strain *Bacillus halotolerans* (BHFHB). The composition of its exopolysaccharides (EPS) was analyzed by gas chromatography. Trees with FH were identified as a distinct morphotype characterized by leaf blade thickness and increased flavonoid content in leaves. It was experimentally demonstrated that this morphotype is characterized by the capacity for flavonoid hypersynthesis. Treatment of leaves with ethephon or bacterial EPS resulted in a 20–30% increase in flavonoid content within four hours. The proactive response, combined with xeromorphic traits, indicates a state of systemic priming. FH formation in this morphotype is proposed to be considered as an individual adaptive strategy, where ethylene-dependent hypersynthesis of flavonoids provides antioxidant protection, biomechanical resistance, and metabolomic adaptation under extreme conditions of mountain ecosystems.

## 1. Introduction

The European beech (*Fagus sylvatica* L.) is one of the most economically significant forest tree species in Central Europe [1]. Beech wood is highly valued, but its quality is often compromised by the formation of false heartwood (FH morphotype). This defect manifests as a noticeable darkening in the central part of the trunk. Although the mechanical properties of the wood remain largely unchanged in the early stages [2], the formation of FH significantly reduces its market value due to strict technological and aesthetic requirements [3].

Despite sustained research, the origin and mechanisms of FH formation are not fully understood [4]. However, tree age is considered the most important factor that increases its probability [5]. Darkening of the central stem wood occurs in living xylem parenchyma cells as a result of their aging and gradual death [3]. According to the Type II heartwood formation model, this process is accompanied by accumulation of precursors of secondary metabolites in ray cells [6].

The classical theory [7] considers broken branches as the main cause of hidden defects, providing entry points for pathogens and oxygen, which, in particular, induces red heartwood formation. However, careful measurements of oxygen in wood have shown that its concentration is sufficient even in trees without heartwood, supporting the hypothesis of specific triggers, possibly microorganisms [3]. Endophytic microorganisms are considered one of the modulators of host metabolism that regulate biochemical pathways underlying wood development, affecting physiological processes related to wood formation [8]. Their role in these processes remains controversial. Recent large-scale studies have demonstrated that tree microbiomes show a distinct split of endophytic communities between heartwood and sapwood tissues [8]. The antimicrobial properties of plant metabolites and the specific environment of the stem (low availability of readily digestible substrates, high CO_2_ concentration, and localized hypoxia) restrain uncontrolled growth of endophytic microorganisms [7,8]. At the same time, due to strict internal habitat filtering, the role of surrounding soil communities in shaping the wood microbiome has proved minor [8]. Plant colonization by microorganisms can occur not only through roots but also through leaves, followed by their spread throughout all tissues and organs [9], with plants being able to regulate this pathway of microbial dissemination and microbiome formation [10]. Polyphenolic compounds, particularly flavonoids, can participate in cell wall modifications and actively influence the mobility and viability of microorganisms in tissues [11,12], which may explain the high concentration of flavonoids along vascular bundles [13].

Recent comprehensive studies support the role of plant growth-promoting bacteria (PGPB), particularly those of the genus *Bacillus*, in enhancing phenylpropanoid synthesis, which leads to several-fold increases in phenolic and catechin content, mitigating the effects of abiotic stress [14]. However, the problem of interaction between the plant organism and endophytes lies in the fact that during prolonged stress, as well as under adverse environmental conditions, even mutualistic endophytes may negatively affect the plant and trigger host defense responses due to shifts in their metabolic activity and modifications in structural components. Under extended drought, some endophytic bacteria synthesize protective exopolysaccharides EPS [15]. EPS molecules and bacterial biofilms in plant tissues are potential elicitors, which may be recognized by morphotypes particularly sensitive to them as specific microbe-associated molecular patterns (MAMPs) [16]. The perception and transduction of MAMP-derived signals within the plant organism activate defense responses [17,18]. In tissues, the level of reactive oxygen species (ROS) and oxidase activity increases [19], stress hormones such as ethylene are produced, and the production of stress proteins and secondary metabolites, including catechins and other compounds with antimicrobial properties, is enhanced [20]. These compounds with antimicrobial properties can destroy biofilms and inhibit quorum sensing in bacteria [12]. Under stress conditions, flavonoids, particularly catechins, polymerize through the formation of reactive quinones via oxidation, resulting in the accumulation of insoluble, melanin-like dark products within parenchyma cells [3,6]. Over time, in xylem zones with elevated polyphenol content, it may lead to the formation of FH. From the raw material perspective, this is considered a defect [4,21]. However, for the tree, such transformation of the internal environment is primarily a matter of the microbiome [22].

Despite growing evidence linking endophytes to host defense and phenolic metabolism, it remains unclear whether bacterial metabolites directly modulate the biochemical pathways associated with FH formation and whether these responses depend on the inherent susceptibility of different *Fagus sylvatica* L. morphotypes to FH development. These directions are essential for advancing our understanding of the biological mechanisms underlying FH formation. We test the following hypotheses: (i) within populations of *Fagus sylvatica* L., there are specific morphotypes highly sensitive to ethylene and bacterial EPS elicitors; (ii) under the influence of bacterial elicitors and hormonal stimuli, phenolic synthesis is hyperactivated in these sensitive morphotypes, directly contributing to accelerated FH formation.

## 2. Results

**Figure 1:**
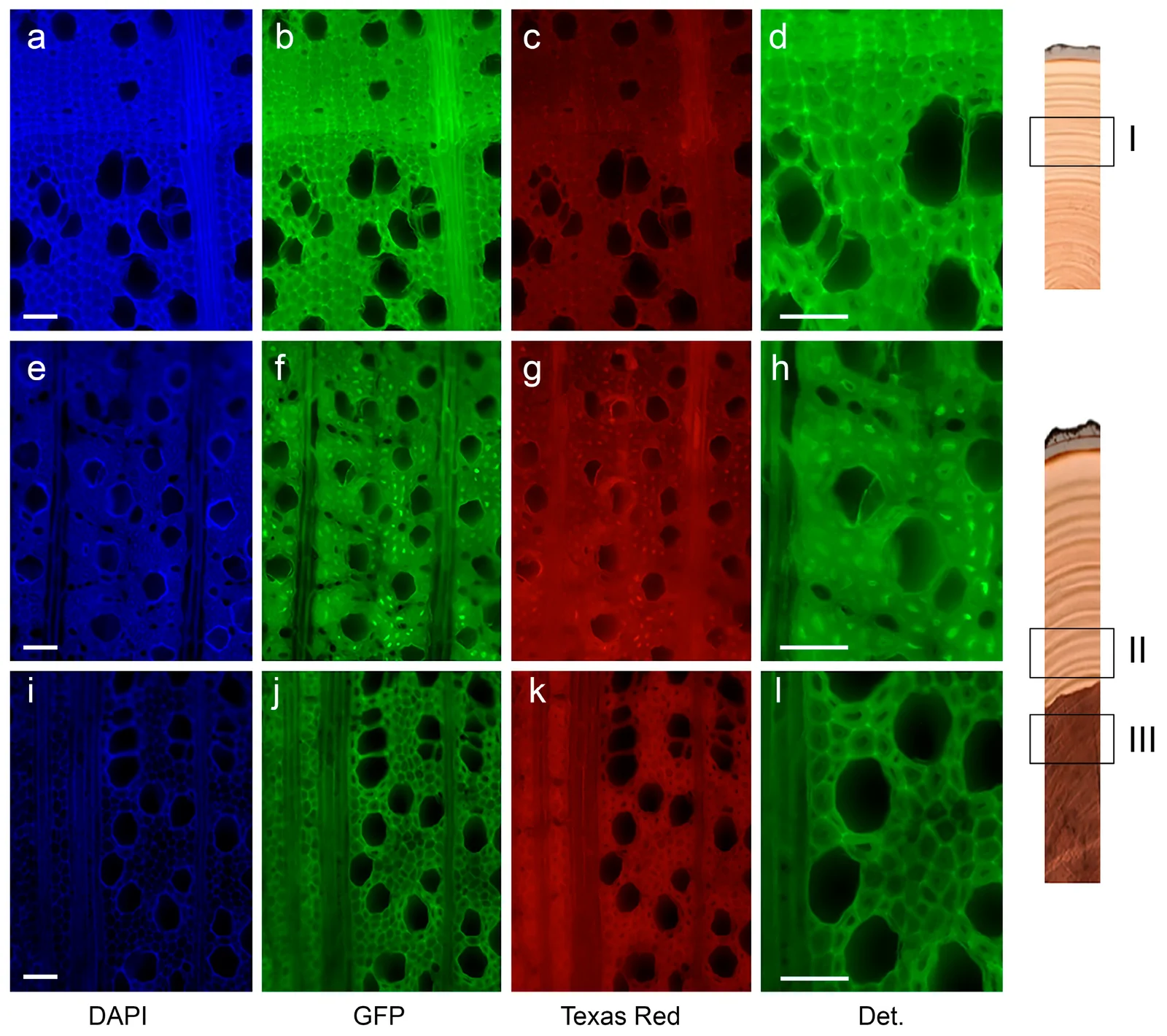
Epifluorescence microscopy of cross sections of *Fagus sylvatica* L. wood: (**a**–**d**) Wood of a plant without signs of FH formation (I); (**e**–**h**) border zone of wood (II) with FH; (**i**–**l**) formed FH (III); (**d**,**h**,**l**) a magnified detail of libriform fibres (GFP); DAPI channel (λem = 447/60 nm); GFP channel (λem = 524/24 nm); Texas Red channel (λem = 628/32 nm); scale bar in all panels represents 50 µm.

FH in *Fagus sylvatica* L. is easily visible on a stem cross-section. In our studies, darkening of the central part of the stem wood had a distinct border (Figure 1). At the tissue level, epifluorescence microscopy revealed spatial phytochemical differentiation of xylem elements by the character and intensity of luminescence. In the xylem (Figure 1, section I) of trees without signs of FH morphotype, the cell walls of vessels and libriform fibers showed moderate and uniform autofluorescence in the blue, green, and red spectra (Figure 1a–d). The surface of primary cell walls showed bright autofluorescence in the blue and green spectra (Figure 1a–c), while the cavities of xylem elements were free of fluorescent inclusions. Cells of radial rays and xylem parenchyma displayed moderate fluorescence intensity. Their cell walls were thickened in the late wood zone. Radial ray cells of early wood were thin-walled, narrow, and laterally elongated. The internal content of living libriform cells was distinguished against the xylem background by low fluorescence, indicating the absence of optically active metabolites.

In the zone adjacent to false heartwood formation (Figure 1 section II), the character and intensity of fluorescence differed markedly from the norm. The cell walls of libriform fibers and vessels showed lower fluorescence compared with the standard (Figure 1f,g), which may indicate light-absorbing activity of cell wall components. Detailing of the fiber structure (Figure 1h) confirmed reduced fluorescence directly in the secondary cell wall. In early xylem, an increased number of small-diameter vessels was noted. At the same time, a distinctive feature of this xylem zone was the presence of numerous xylem parenchyma cells with bright autofluorescent products inside protoplasts, as well as living libriform fibers. The contents of these cells were most clearly visualized in the green spectrum and less clearly in the red (Figure 1f,g). Multirow radial rays of the xylem appeared as darkened linear structures. The degree of darkening of protoplast contents increased centripetally, with enhanced light-absorbing activity at the boundary of the FH.

A similar effect was observed in the protoplasts of xylem parenchyma. In the red spectrum, radial ray cells and xylem elements retained the capacity for intense autofluorescence (Figure 1g). A characteristic feature of tracheids at the border with FH was the bright luminescence of inner cell walls against the background of overall fluorescence quenching in the blue spectrum (Figure 1e). In the FH zone (Figure 1, section III), the tendency towards light-absorbing activity of radial ray cells and secondary cell walls of fibers was intensified across all channels studied. The optically active contents of parenchyma protoplasts and living libriform fibers in this zone lost their ability to fluoresce (Figure 1j,l). Under light microscopy, the intracellular contents were visualized as massive aggregations of amorphous brown material. At the same time, the brightness of the fluorescence of lignified primary cell walls remained unchanged.

Thus, epifluorescence analysis of xylem in the FH zone revealed signs of specific modification of secondary cell walls, ranging from reduced to ceased autofluorescence intensity in certain elements. Spatial heterogeneity was revealed in the distribution of optically active polyphenolic compounds within xylem parenchyma, with their concentration in the zone of FH formation. The synthesis, distribution, and systemic localization of these compounds in xylem elements are likely to be manifested in other metabolically active centers of the plant organism.

### 2.1. Morphometric Characteristics of European Beech Leaves with Signs of False Heartwood

Analysis of morphometric parameters of leaf blades revealed differences between the studied morphotypes of *Fagus sylvatica* L. (Table 1). It was found that trees with signs of FH were characterized by a decrease in the perimeter and area of leaf blades.

However, high intra-group variability of linear parameters did not allow these changes to be considered statistically significant (for leaf area, *p* = 0.145). At the same time, the leaf blade thickness (LLT) in the FH morphotype was significantly greater by 19.15% (*p* < 0.001). Significant differences were also established for the Circularity index (Circ, *p* = 0.026) and the Dissection index (DI, *p* = 0.040). Taken together with the marked thickening of the mesophyll and the midrib, these differences are caused by the spatial deformation of the leaf blade (formation of a corrugated surface). The remaining indices describing the general proportions of the leaf blade showed morphogenetic stability in the studied trees.

**Figure 2:**
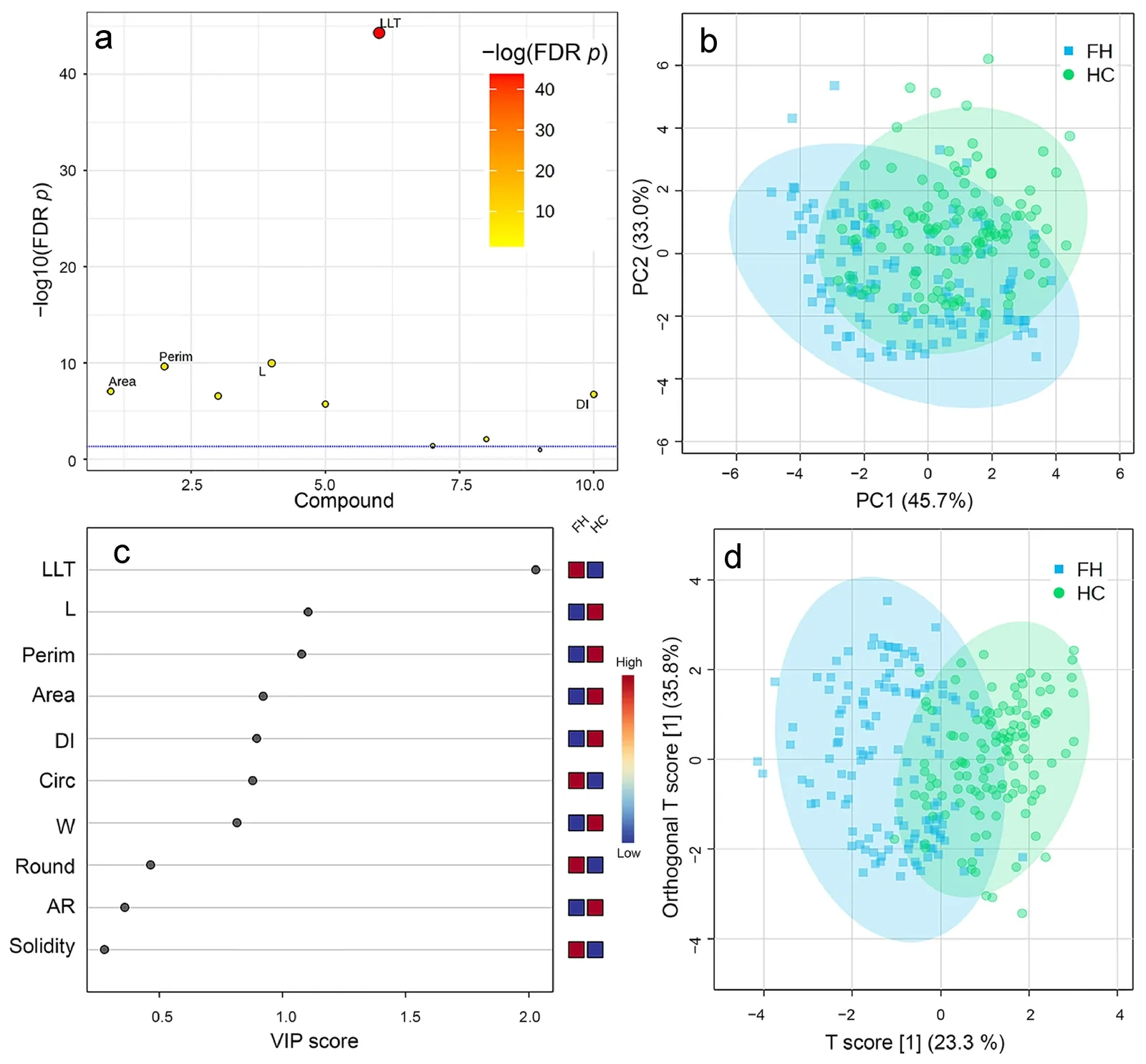
Results of multivariate statistical analysis of morphometric parameters of *Fagus sylvatica* L. leaves: (**a**) plot of morphometric trait distribution in coordinates of significance level (–log_10_ (FDR.*p*)) and effect size; (**b**) scatter plots from principal component analysis (PCA); (**c**) VIP scores and heatmap: diagram of variable contribution to the model (variable importance in projection) with heatmap of relative parameter values for FH—false heartwood morphotype and HC—healthy control morphotype; (**d**) OPLS-DA score plot: scatter diagram in the plane of orthogonal projections with coordinates T score (23.3%) and orthogonal T score (35.8%).

To identify the most informative morphological traits, principal component analysis (PCA) was additionally applied. The first two principal components accounted for 78.7% of the total data variance (PC1—45.7%, PC2—33%), demonstrating sufficient representativeness of the selected morphometric leaf traits. The plot showed separation between the control group of trees with healthy control (HC morphotype) wood and trees with FH. The main separation of groups occurred along the first principal component. The FH cluster was shifted to the left part of the plot, while the HC cluster tended towards the upper right part. Since the PC1 axis explained the largest share of variation (45.7%), this confirms the presence of significant differences in leaf morphology associated with the physiological state of the trees. On the plot, the confidence interval ellipses showed an overlap zone, but the centroids of the clusters were spatially separated in the principal component space. As the vectors of size parameters (Area, L, W) were directed towards the HC cluster and the vector of LLT towards the FH cluster, the cloud of blue points in the left part of the plot reflects the tendency of trees with false heartwood towards xeromorphization of leaf structure. This is also supported by orthogonal partial least squares discriminant analysis (OPLS-DA). The overall discriminant model demonstrated predictive ability and reliability acceptable for drawing valid conclusions (R2Y = 0.233, Q2 = 0.392), which is confirmed by spatial separation of the studied morphotypes along the first orthogonal axis (Figure 2b).

The OPLS-DA discriminant analysis model confirmed discrimination between FH and HC morphotypes. Variable importance in projection (VIP) analysis identified LLT as the key determinant clearly distinguishing the group of trees with FH. This parameter showed the highest discriminatory potential (VIP > 2.0; Figure 2c). The validity of this marker is supported by the low adjusted significance level (FDR *p* < 0.001, Figure 2a).

### 2.2. Isolation and Identification of Endophytic Bacteria from the Discolored Part of European Beech Wood

**Figure 3:**
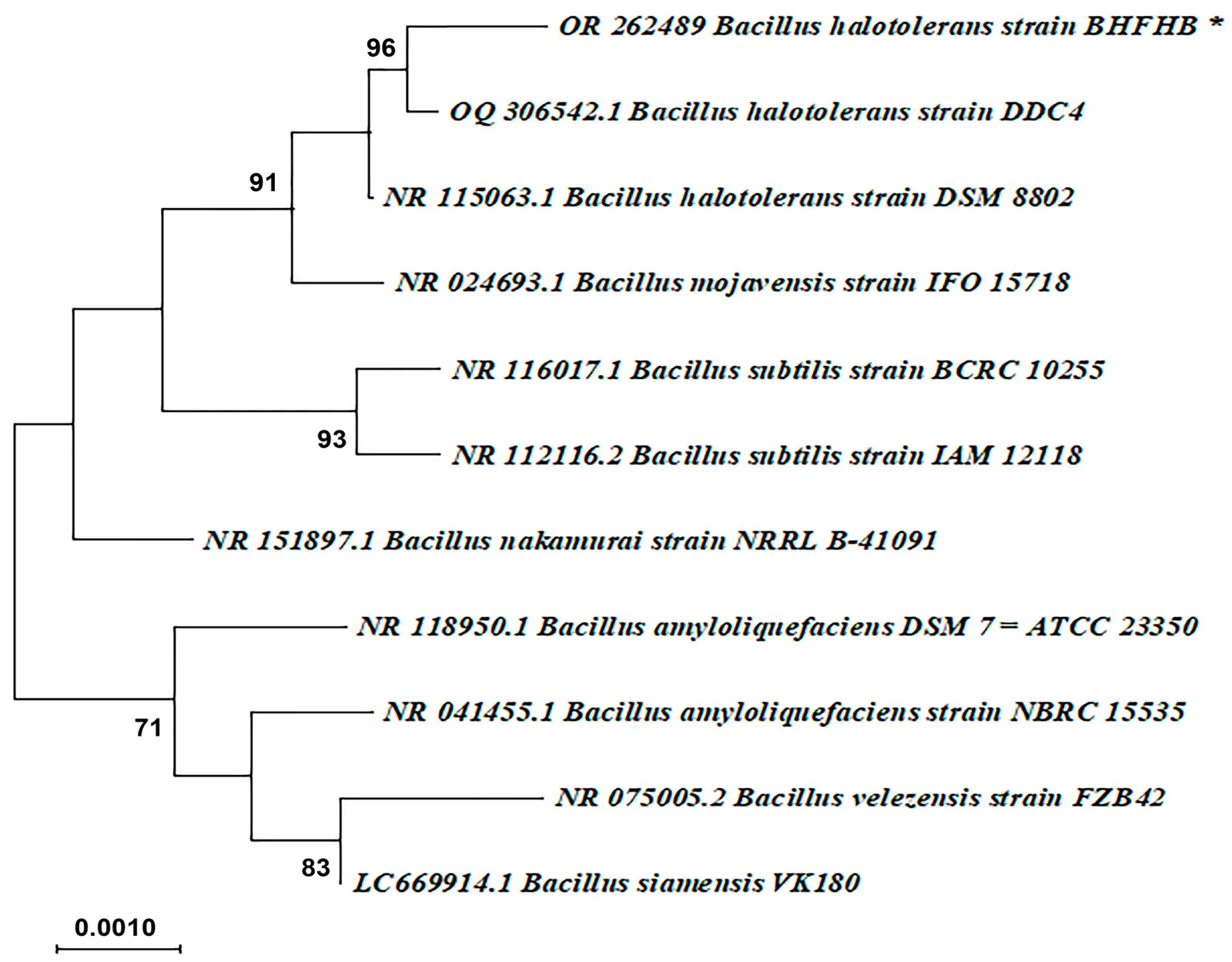
Dendrogram of genetic similarity between the typical strains of the genus *Bacillus* and *Bacillus halotolerans* BHFHB (marked with an asterisk), constructed on the basis of 16S rRNA gene sequences using the neighbor-joining method with Kimura’s two-parameter model.

The ability of endophytes to induce a priming effect in trees, enhancing metabolic activity accompanied by the synthesis of phenolic compounds, suggests their gradual accumulation and deposition in zones of non-functional heartwood. It may be assumed that under certain conditions, this accumulation can undergo slow or rapid oxidation and polymerization, leading to FH formation. To clarify the possible involvement of microorganisms in such processes, endophytic bacteria and micromycetes were isolated from the boundary zone between light and darkened wood. From the obtained prokaryotic cultures, a Gram-positive spore-forming bacterium was selected, which demonstrated significant antifungal activity and the ability to produce EPS. For species identification of the strain, molecular-genetic and phylogenetic analyses were performed, including sequencing of the 16S rRNA gene and comparison of the obtained sequence with those available in GenBank. Sequencing yielded a fragment of 1454 bp from *Bacillus halotolerans* BHFHB, showing 99.86% similarity to BHFHB LMG 2247. Based on the 16S rRNA gene sequences of the studied strain and other species of the genus *Bacillus*, a phylogenetic dendrogram was constructed, reflecting the degree of genetic similarity among different *Bacillus* species (Figure 3).

**Figure 4:**
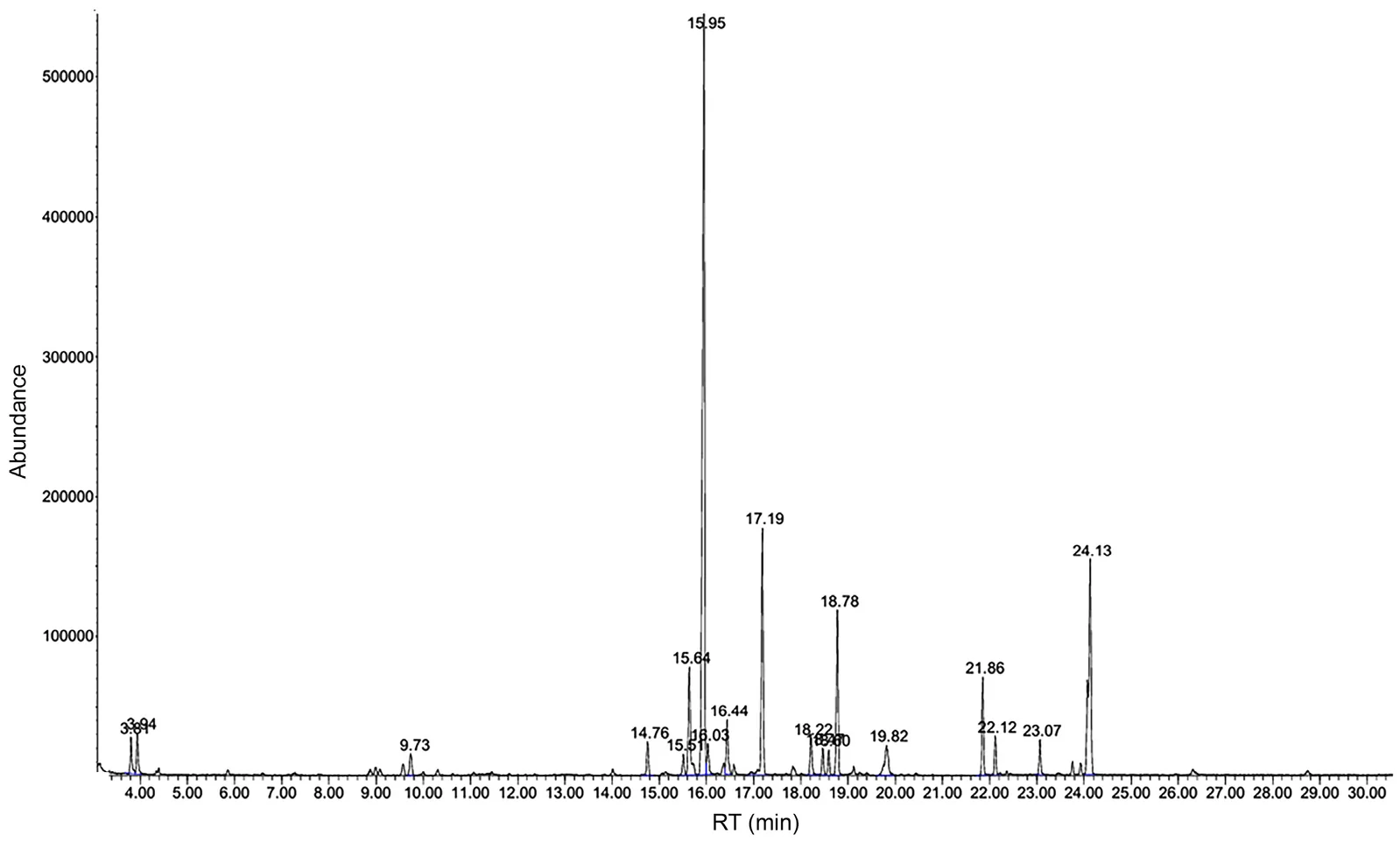
Gas chromatography of components in the exopolysaccharide of *Bacillus halotolerans* BHFHB.

The isolated strain BHFHB, when cultivated in liquid LB medium supplemented with sucrose, produced a substantial amount of EPS. Considering the important role of EPS in the adaptation of this endophytic bacterium to extreme conditions, and the potential of polysaccharide components to influence the plant, its chemical composition was analyzed. Gas chromatography revealed three main components in the heteropolysaccharide: glucose, mannose, and galactose (Figure 4).

After calculating the signal intensities on the chromatogram, the relative content and ratios of monosaccharides in the composition of EPS-BHFHB were determined (Table 2).

The presence of mannose and galactose in the EPS composition confirmed the potential ability of oligosaccharides or monosaccharides to function as MAMPs. Plants can recognize mannose and galactose molecules through lectins sensitive to them, which may be accompanied by activation of defence signalling pathways and protective responses. In this regard, it was hypothesized that under unfavorable conditions for the endophytic bacterium, active EPS synthesis may increase the amount of potential MAMPs in the apoplast. If the plant is highly sensitive, it may perceive them as a bacterial invasion and trigger induced resistance responses.

### 2.3. Tissue Specificity and Hyperactivation of Autofluorescence in Response to EPS

**Figure 5:**
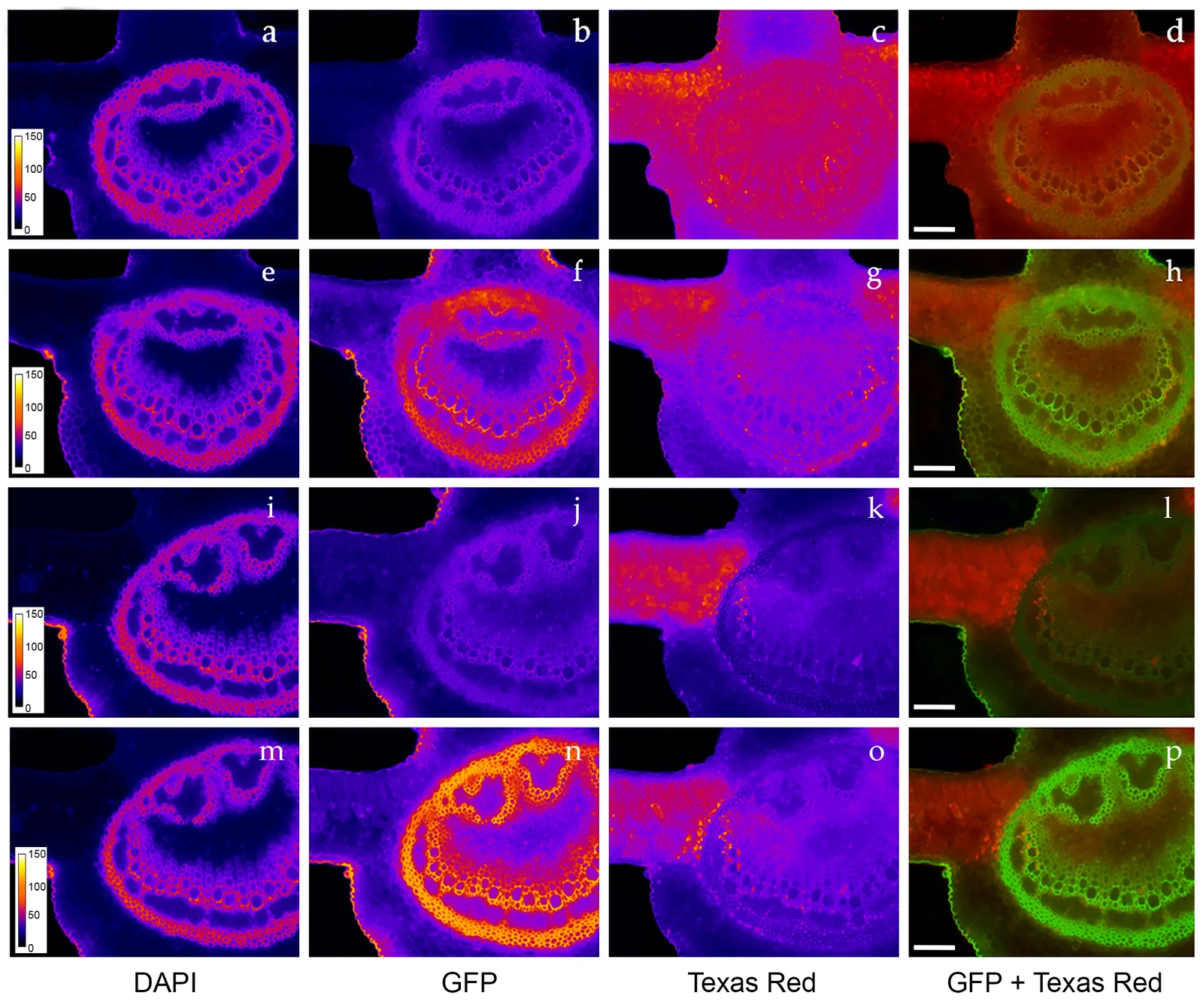
EPS-BHFHB induced changes in the autofluorescence of *Fagus sylvatica* L. leaf tissues. Visual analysis (Panels **a**–**p**): Representative fluorescence intensity heatmaps and merged fluorescence images of leaf cross-sections before and after treatment with bacterial exopolysaccharide EPS; (**a**,**e**,**i**,**m**): DAPI channel (λem = 447/60 nm); (**b**,**f**,**j**,**n**): GFP channel (λem = 524/24 nm); (**c**,**g**,**k**,**o**): Texas Red channel (λem = 628/32 nm); (**d**,**h**,**l**,**p**): merged images (GFP + Texas Red); scale bar—50 µm.

Multichannel fluorescence imaging of transverse sections of the central vein of *Fagus sylvatica* L. leaves revealed substantial changes in the optical properties of tissues after treatment with a 0.1% aqueous solution of EPS-BHFHB. Epifluorescence analysis confirmed the influence of the bacterial heteropolysaccharide on the intensity of fluorescence in mechanical and conductive tissues. The most pronounced signal enhancement was observed in the cell walls of sclerenchyma (Scl) and xylem vessels (Xyl), clearly visualized in the DAPI and GFP channels (Figure 5a–p).

**Figure 6:**
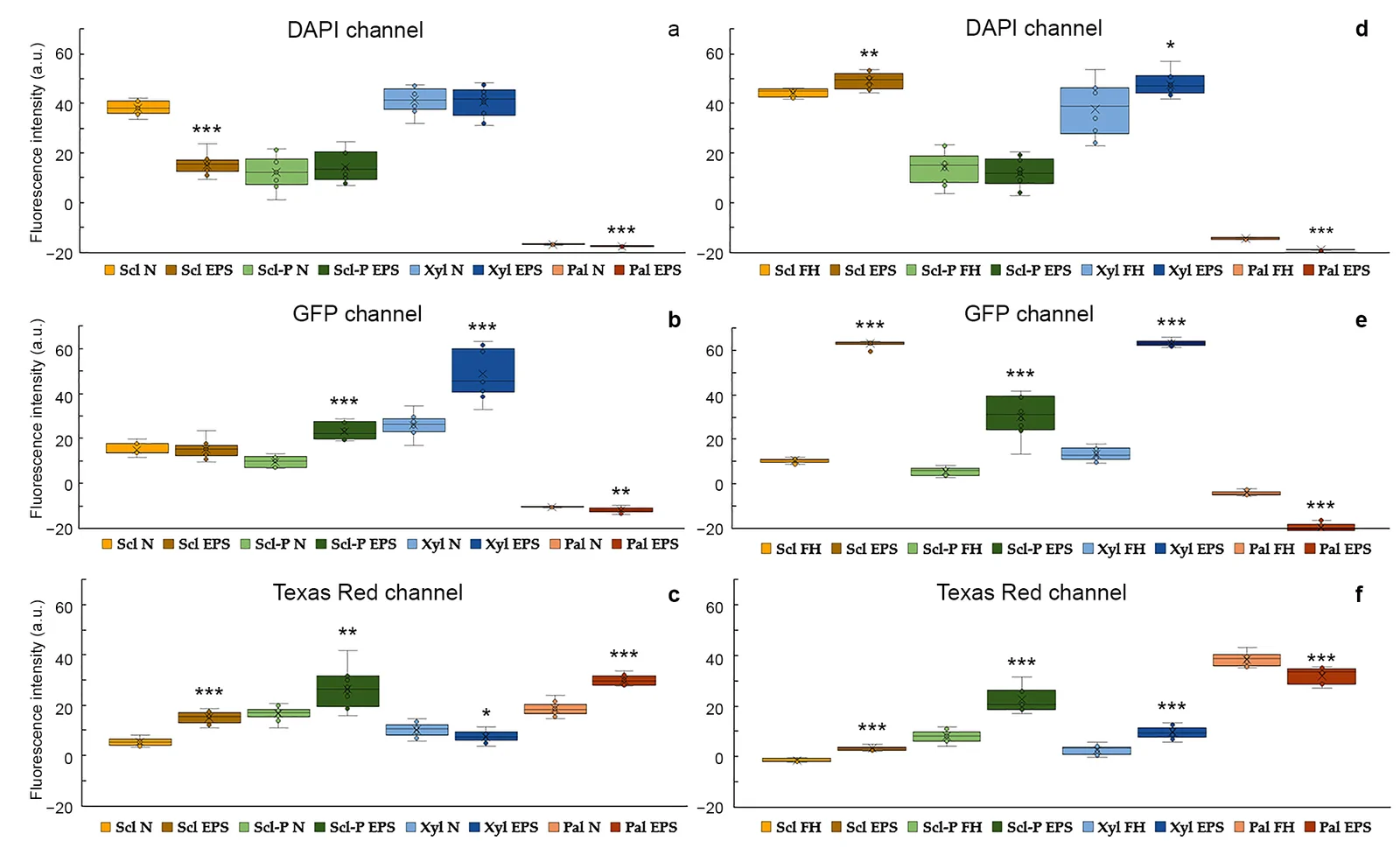
Quantitative analysis of the autofluorescence of *Fagus sylvatica* L. leaf tissues: Box plots representing fluorescence intensity across four tissue types: Scl (sclerenchyma cell walls), Scl-P (sclerenchyma protoplasts), Xyl (xylem), and Pal (palisade parenchyma); Left plots (**a**–**c**): HC—healthy control morphotype response (N); right plots (**d**–**f**): FH: false heartwood morphotype response. Data are expressed as mean fluorescence intensity in arbitrary units (a.u.). Statistical significance (* *p* < 0.05, ** *p* < 0.01, *** *p* < 0.001) indicates differences between Control and EPS-BHFHB-treated samples.

Overlaying the red and green channels (merged images) enabled visualization of differences in active fluorescence signals in sclerenchyma cell protoplasts (Scl-P), particularly evident in the central veins of leaves from trees with FH (Figure 5l,p). Statistical data processing confirmed the phenomenon of tissue hypersensitivity in leaves of FH trees by signal level. In FH leaves, EPS treatment caused a multiple increase in fluorescence intensity in sclerenchyma (Scl) and xylem (Xyl) in the green channel (Figure 6e).

At the same time, in the red channel (Texas Red), a decrease in signal intensity was observed in the mesophyll and in the pith parenchyma of the central vein (Figure 6c,f). The fluorescence intensity in the xylem parenchyma remained unchanged. Fluorescence of lignified tissues (Scl, Xyl) in the blue spectrum also tended to increase under the influence of EPS-BHFHB, with trees showing FH exhibiting both a higher baseline signal level and a greater increase (Figure 6a,d).

**Figure 7:**
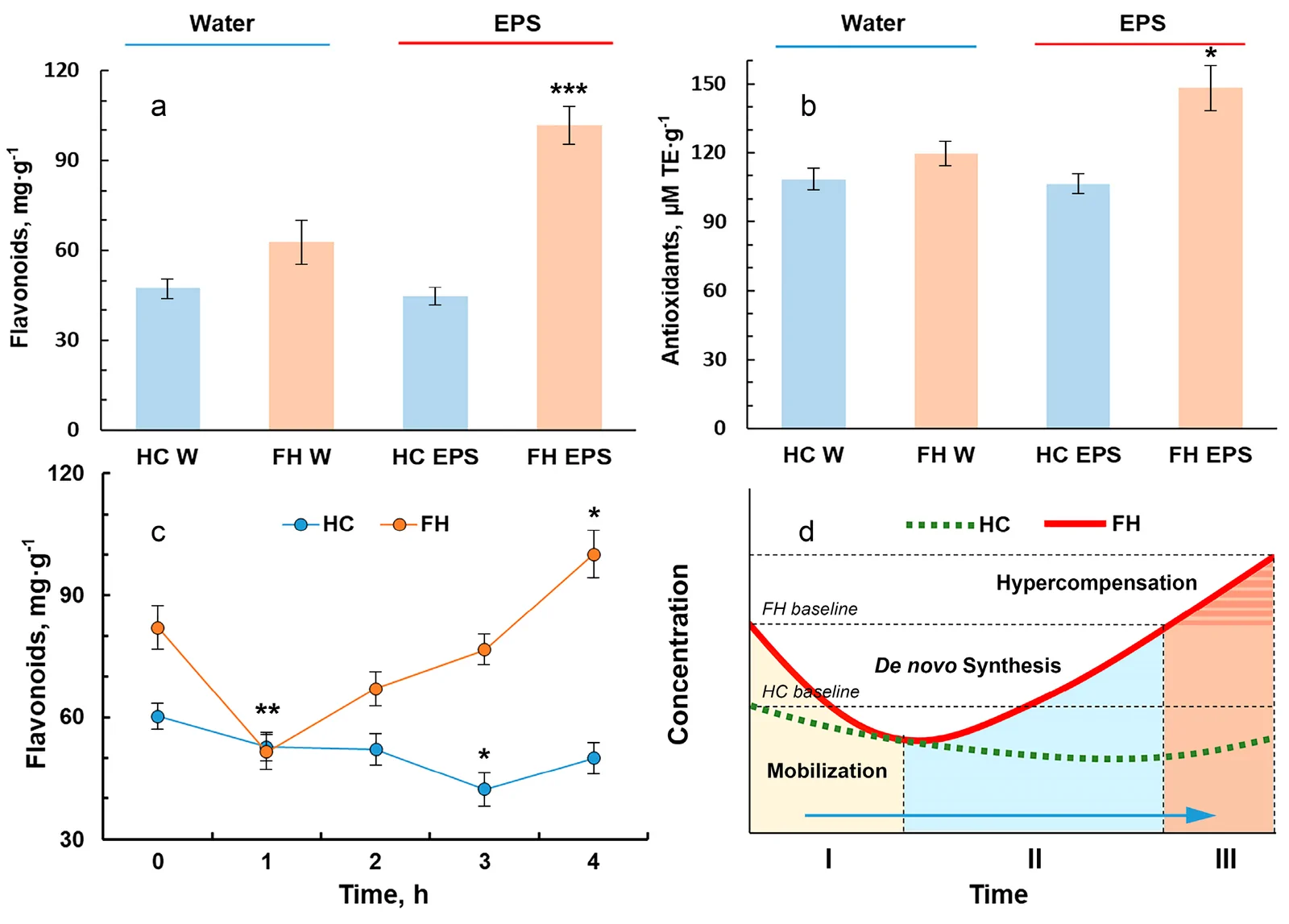
Influence of EPS-BHFHB of *Bacillus halotolerans* BHFHB and ethephon on secondary metabolism of *Fagus sylvatica* L. leaves: (**a**) flavonoid content after treatment; (**b**) antioxidant activity of phenols; (**c**) dynamics of flavonoid content during 4 h after ethephon treatment; (**d**) graphical model of the kinetics of physiological responses of model morphotypes to ethylene stimulus: (I) mobilization: active expenditure of available constitutive flavonoids in defense reactions; (II) de novo synthesis: initiation of flavonoid synthesis followed by their use in induced resistance reactions (solid red line for FH phenotype, dashed green line for control); (III) hypercompensation: exceeding the baseline level of flavonoids. The statistical significance of differences for histograms (**a**,**b**) and time-course dynamics (**c**) was determined via pairwise comparisons using the Mann–Whitney U test (or Student’s *t*-test where appropriate) relative to the corresponding control groups. Significance levels are indicated as * *p* < 0.05, ** *p* < 0.01, and *** *p* < 0.001.

To test the hypothesis of a systemic nature of histochemical reactions and the role of polyphenols in them, the dynamics of flavonoid content in beech leaves after treatment with the bacterial heteropolysaccharide was analyzed. Application of a 0.1% solution of EPS-BHFHB to leaves showed an increase in flavonoid content in leaves of the trees with FH by 63.3% after 6 h. Their concentration in leaf tissues reached 87.9–116.7 mg/g of dry weight (Figure 7a). It was accompanied by a significant increase in the total antioxidant activity of phenolic compounds (up to 148.2 ± 9.9 µmol Trolox equivalents per gram of dry weight (µmol TE/g)), indicating the functional importance of the synthesized metabolites and a change in the redox status of tissues (Figure 7b). In trees with HC, leaf treatment with EPS-BHFHB resulted in statistically insignificant differences in flavonoid content and antioxidant activity compared with the control.

The tissue response to bacterial EPS revealed the presence of trees within the population prone to developing induced plant resistance. Since FH morphotype trees, by their flavonoid synthesis activity as well as morphological and histochemical traits, exhibited features of systemic priming, it was important to clarify which basic regulatory mechanisms might be involved in the hypersensitivity reactions of the FH morphotype to EPS-BHFHB. To this end, in leaf tissues, an increase in ethylene levels was modeled using ethephon (an ethylene donor), as one of the modulators of xeromorphogenesis and flavonoid synthesis.

Using the criteria recommended by Knoke (2003) [5], two model *Fagus sylvatica* L. trees were selected. To avoid the influence of traumatic factors, leaves on the trees were treated with a 10 mM solution of ethephon without taking them from the shoots. Analysis of the dynamics of total flavonoid content showed that in the leaves of both model trees, regardless of classification, a decrease in flavonoid content was observed during the first hour after ethephon treatment (Figure 7c). Differences in responses between the model trees appeared during the subsequent three hours. In the leaves of HC morphotype plants, a gradual decrease in flavonoid content was observed, reaching a minimum (42.2 mg/g) three hours after treatment. In contrast, in the leaves of FH morphotype plants, a rapid accumulation of flavonoids occurred. Overall, within four hours, the total flavonoid pool in the leaves of FH trees increased by 21.9% compared with the initial level. The significant difference in responses between the model plants and the similarity of FH morphotype trees’ reactions to treatment with the endophyte EPS-BHFHB and ethylene confirmed the potential predisposition of FH trees to flavonoid hypersynthesis under biotic and abiotic stress conditions.

Based on the dynamics of flavonoid content in leaves under ethephon treatment (Figure 7c), a conceptual model of morphotype metabolic responsiveness was developed (Figure 7d). The model distinguishes three distinct functional phases. In Phase I (0–1 h), both morphotypes exhibit a rapid decline in flavonoid content, reflecting the utilization of constitutive pools for initial defense responses. In Phase II (1–3 h), a clear difference between the morphotypes is observed: the HC morphotype maintains the trend of using flavonoid reserves, whereas the FH morphotype demonstrates signaling competence, initiating active de novo biosynthesis. By the end of this phase, the de novo synthesized flavonoids fully restore the reserve. Finally, Phase III (3–4 h) represents a state of metabolic hypercompensation specific to the FH morphotype, characterized by a rapid surge in flavonoid content well above the initial baseline levels.

## 3. Discussion

This study revealed a functional relationship between the xeromorphic structure of leaves of *Fagus sylvatica* L. and their increased sensitivity to bacterial heteropolysaccharides of BHFHB and to ethylene. This feature is characteristic of trees aged approximately 80 years with signs of FH formation. The obtained data suggest that heartwood transformation is preceded by a transition of trees into a state of systemic priming, which occurs under the synergistic action of abiotic factors and as a result of plant–microbe interactions.

### 3.1. Relationship Between Crown Architecture, Hydraulic Conductance, and Leaf Structure

Phytohormonal regulation of growth processes and long-term plant adaptation to stress factors directly correlate with crown architecture and overall tree habit. In particular, hormonal imbalance induced by chronic moisture deficit or other environmental factors can weaken apical dominance. This directly shapes the morphology of the main stem and lateral branches, creating predispositions for the formation of forks and massive branches [4,5]. Within these junction zones, narrower and shorter vessels can significantly reduce hydraulic conductance [14,23]. During peak transpiration hours, this localized flow restriction causes a drop in water potential, intensifying tissue moisture deficit and promoting the development of xeromorphic leaf features. Consequently, the predictive model by Knoke (2003) [5], developed for diagnosing FH, logically identifies stem forking as one of the most critical predictors of this wood defect.

This structural feature of the trunk, along with long scaffold branches, can complicate the regulation of the tree’s water balance [24] and create prerequisites for additional gravitational loading on tissues, which, in combination with moisture deficit, enhances ethylene synthesis in the reaction part of wood [25]. The rapid increase in trunk diameter, also considered in the Knoke model, leads to an increased volume of physiologically inactive xylem and results from tissue responses to phytohormonal stimuli. Ethylene is known to be a key regulator of heartwood formation [26]. In *Dalbergia odorifera*, it stimulates the biosynthesis of secondary metabolites specifically in heartwood tissues and significantly increases their area within branches [27].

Our epifluorescence microscopy data (Figure 1) provide visual evidence of histochemical and functional changes in the xylem of a tree with a false core. The pronounced spatial phytochemical differentiation observed in xylem sections highlights a progressive metabolic shift from the sapwood toward the FH boundary. In the HC morphotype (Figure 1, section I), uniform moderate autofluorescence of vessels and libriform cell walls reflects the baseline state of structural lignification, under which the tissue is optimized exclusively for upward hydraulic transport and lacks active accumulation of polyphenols.

In wood with a FH, within the transition zone (Figure 1, section II), the protoplasts of living libriform fibers, xylem parenchyma, and radial rays are saturated with products showing bright autofluorescence in the green and red spectra. While autofluorescence alone cannot uniquely identify specific compounds, such an emission pattern inside protoplasts is typically associated with flavonoids, their conjugates, and oxidized quinones, alongside potential contributions from other phenolic derivatives [28]. This localized filling of cells with polyphenols and their conjugates with cinnamic acids confirms the tissue priming effect in wood and represents an important reserve for strengthening and protecting the conductive system. With the participation of peroxidases, polyphenols can integrate into the cell wall matrix of tracheids and libriform fibers [29].

This process is clearly visible in the FH zone of wood (Figure 1, section III). The complete loss of protoplast fluorescence and the appearance of massive amorphous brown cell content represent the final, irreversible stage of this defensive strategy. The spatial histochemical heterogeneity of tissues confirms that the transition zone acts as a dynamic metabolic front. The synthesis, distribution, and systemic localization of these protective polyphenols in elements of the stem xylem suggest their link with other active centers of the plant. Gradually restricting the total volume of physiologically active sapwood, this atypical modification of *Fagus sylvatica* L. wood establishes a long-term hydraulic constraint, which leads to a parallel, phytohormonally regulated general xeromorphization of the woody plant.

On the whole, the variability in linear parameters of leaves of the studied morphotypes of *Fagus sylvatica* L. in the forest stands of the Bukovinian Pre-Carpathians (Ukraine) can be regarded as an indicator of the species’ phenotypic ecological plasticity, explained by substantial intrapopulation variability [30,31]. According to the results of multivariate analysis, OPLS–DA on the studied trees divided by the criterion of presence or absence of FH (established after felling), the key discriminant was LLT (VIP > 2.0, FDR.*p* < 0.001). Leaves with signs of FH morphotype were characterized by a smaller surface area, but a thicker leaf blade and a more developed palisade parenchyma. A well-developed mesophyll and a system of conductive and mechanical tissues are signs of organ xeromorphization, which is regulated phytohormonally [32]. Considering that the studied trees grew under identical environmental conditions, the predisposition of the FH-morphotype toward xeromorphic features may indicate differences in their metabolism and priming of the plant with an increase in polyphenol synthesis.

### 3.2. Foliar Flavonoid Dynamics in Response to Ethylene Stimulus

Reduction in leaf area is also a consequence of either ethylene overproduction or plant hypersensitivity to it [33]. Signaling of this hormone subsequently promotes catechin accumulation [34]. Under the influence of ethylene, flavonoids accumulate within both guard cells [35] and leaf parenchyma vacuoles. In the mesophyll, enhanced catechin accumulation provides crucial buffering against the increased production of ROS, which accumulate in tissues under drought stress [25,36,37], thereby supporting the maintenance of cellular integrity and turgor even under conditions of chronic vessel cavitation [36].

In our experiment, after treatment of leaves with ethephon, a significant decrease in flavonoid content was observed during the first hour, regardless of morphotype (Figure 7c). However, during the following three hours, the dynamics of flavonoid content differed between morphotypes. In the HC morphotype, the tendency toward a gradual decrease persisted, whereas in the FH morphotype leaves, the total flavonoid pool began to increase two hours after treatment. By the fourth hour, their total content increased by 21.9%. In contrast to the unreactive HC morphotype, a similar dynamics was observed when FH leaves were treated with an exopolysaccharide from an endophytic bacterium. Taking into account the differences in the action rate of the direct hormonal stimulus (4 h) versus the macromolecular elicitor, after 6 h, their flavonoid content increased by 38.7%. Such responsiveness of flavonoid biosynthesis indicates a specific tissue competence for ethylene signaling and an enhanced capacity for rapid mobilization of enzymatic complexes in trees with FH.

### 3.3. Enzymatic Regulation and Spatial Organization of Flavonoid Biosynthesis

The reduction in the total soluble flavonoid pool within the first hour post-treatment with ethephon and EPS-BHFHB is consistent with the results of epifluorescence analysis. Thus, after treatment of freshly cut leaf blade tissues with the bacterial EPS BHFHB, the fluorescence of the cuticle as well as of xylem and sclerenchyma cell walls increased markedly within 15 min (Figure 5). The baseline optical signal of vascular bundle tissues in the blue and green channels in the FH morphotype was initially 20–30% higher, which provides visual evidence of priming. Enhanced autofluorescence of lignified elements in leaves under the action of EPS-BHFHB may be interpreted as a response reaction of induced resistance. The increase in optical signal after EPS-BHFHB treatment of sections may be explained by copolymerization of flavonoid components into the structure of secondary cell walls with the formation of flavonolignins mediated by peroxidases [29].

In *Fagus sylvatica* L. leaves, (+)-catechin, (–)-epicatechin, the aglycones quercetin and kaempferol, and their conjugates were identified [36]. Among the products of the phenylpropanoid pathway for lignin synthesis, p-coumaric, ferulic, and caffeic acids predominate. Complex acylated flavonols, including quercetin-O-p-coumaroyl-feruloyl-deoxyhexoside and kaempferol-O-di-p-coumaroyl-deoxyhexoside, were also detected [38]. The structure of these molecules suggests their ability to integrate into the cell wall matrix. This could account for the significant enhancement of leaf cell wall autofluorescence observed in the DAPI and GFP channels (Figure 5) following EPS treatment. Since apigenin and its conjugates (flavones) do not accumulate in beech leaves, there is reason to hypothesize that the main metabolic flux initiated from p-coumaroyl-CoA is routed sequentially via CHS and CHI to naringenin, and then directed via F3H to FLS for the targeted production of quercetin and kaempferol, subsequently feeding into the synthesis of the flavan-3-ols (+)-catechin and (–)-epicatechin.

Activation of flavonoid biosynthesis as early as two hours is consistent with the classical metabolon model [39,40], in which cinnamate 4-hydroxylase (C4H) acts as an integral membrane anchor on the surface of the endoplasmic reticulum (ER). Together with other membrane-bound cytochrome P450 proteins (F3’H), it serves as a structural hub tethering soluble enzymes (CHS, CHI, and FLS), via direct protein–protein interactions [41]. Formation of such metabolons explains the exceptionally rapid flavonoid biosynthesis, since the spatial organization of the involved enzymes has been optimized for catalytic efficiency [42,43,44]. The observed enhanced basal levels of flavonoids in both leaves and wood further support the potential capacity of the FH morphotype for elevated biosynthesis activity.

The prolonged adaptive response observed in leaves may be mediated via the canonical ethylene signaling pathway through stabilization of the transcription factors EIN3/EIL1, followed by activation of ethylene response factor (ERF) genes [45,46,47]. These factors, upon binding to GCC-box motifs in promoters, induce the expression of structural genes of the phenylpropanoid pathway. Thus, ethylene functions as an initiator of long-term systemic priming [48,49,50]. Under such conditions, a portion of the accumulated flavonoids and their derivatives is integrated into cell wall polymers and oxidized by enzymes to form dark-colored products [29,51].

### 3.4. Plant Responsiveness to Elicitors and Bacterial Exopolysaccharide Effects

Polyphenols, in addition to constitutive resistance, possess pronounced antimicrobial properties. Their active synthesis in leaf and beech xylem tissues inevitably modulates the structure of the plant microbiome [8,52]. Under conditions of a significant amount of toxic polyphenols and chronic abiotic stress, endophytic bacteria, including BHFHB, adapted to localized hypoxia and moisture deficit, form protective EPS [15,53]. EPS actively bind water and, thus, are capable of locally creating an excessive water deficit in parenchyma cells. In turn, monosaccharides (glucose, mannose, and galactose) that are part of EPS can be perceived by the plant as MAMPs [16,18]. In the presence of appropriate lectin receptor kinases (LecRLK) in plant tissues, induced resistance reactions are triggered [54,55,56]. One such mechanism involves the activation of ACS and ACO genes responsible for the synthesis of ethylene and its immediate precursors [57,58]. This receptor-mediated process of activation of secondary metabolism explains the de novo accumulation of flavonoids in the leaf parenchyma after EPS-BHFHB treatment. In the context of false heartwood formation, it is particularly interesting that the metabolic burst observed in the FH morphotype under the action of ethylene or EPS-BHFHB is completely absent in the HC morphotype with normal wood.

Since the described induced resistance reactions may be linked to bacterial metabolites, understanding endophyte functioning and their interaction strategies with the plant requires a broader context. The properties of plant growth-promoting endophytes (PGPEs) need to be tested under diverse conditions. Effects characteristic of endophytes in healthy plants can be quite different under physiological stress. The metabolic shifts in leaves observed under the action of EPS and ethylene allow us to consider them as an early systemic priming of trees. It is highly probable that analogous local reactions to bacterial EPS also take place within the living xylem parenchyma of the trunk. Consequently, year-after-year repeated priming effects can cumulatively initiate the multi-stage process of polyphenol compartmentalization, ultimately resulting in false heartwood formation over successive seasons.

It should be noted that the hypersensitivity of *Fagus sylvatica* L. to bacterial EPS potentially exhibits a distinct ontogenetic dynamic. Preliminary observations from our ongoing work on old-growth beech trees (over 100 years) indicate a potential decrease in the threshold of leaf sensitivity to bacterial EPS regardless of the presence of false heartwood. Although these preliminary trends require further verification, they are consistent with findings regarding the increased susceptibility of old-growth trees to the formation of FH [5,59] and the development of butt heart rot in mature stands [60]. Further studies on the age-related leveling of morphotype differences in sensitivity to bacterial elicitors and ethylene may advance the understanding of the mechanisms underlying the epigenetic control of hypersensitive responses in *Fagus sylvatica* L.

### 3.5. Limitations and Perspectives

While this study reveals new aspects of the biochemical and physiological processes associated with false heartwood formation in beech, certain limitations in the interpretation of results and generalizations should also be acknowledged, primarily related to the small sample size, the use of a model-based approach, the measurement of a single class of secondary metabolites (flavonoids) without identifying individual compounds, and the absence of direct molecular evidence for the proposed ethylene-dependent mechanism. Within the scope of our objectives, the analysis focused on a targeted exploration of plant-microbe interactions and the identification of differences in morphophysiological responses among the studied morphotypes. The ability of strain BHFHB, isolated from the beech wood at the FH boundary, to synthesize a significant amount of EPS containing glucose, mannose, and galactose enabled the identification of a high specific sensitivity of the FH-morphotype to them. At the same time, the question of its sensitivity to EPS from other bacterial species or individual sugars remains open.

Nevertheless, despite the aforementioned limitations, the established morphophysiological features of trees with the false heartwood phenotype form a reliable baseline and open broad perspectives for further comprehensive ecophysiological and molecular-genetic investigations, taking into account population variability, environmental conditions, and plant age.

## 4. Materials and Methods

### 4.1. Place of Sampling Leaves

**Figure 8:**
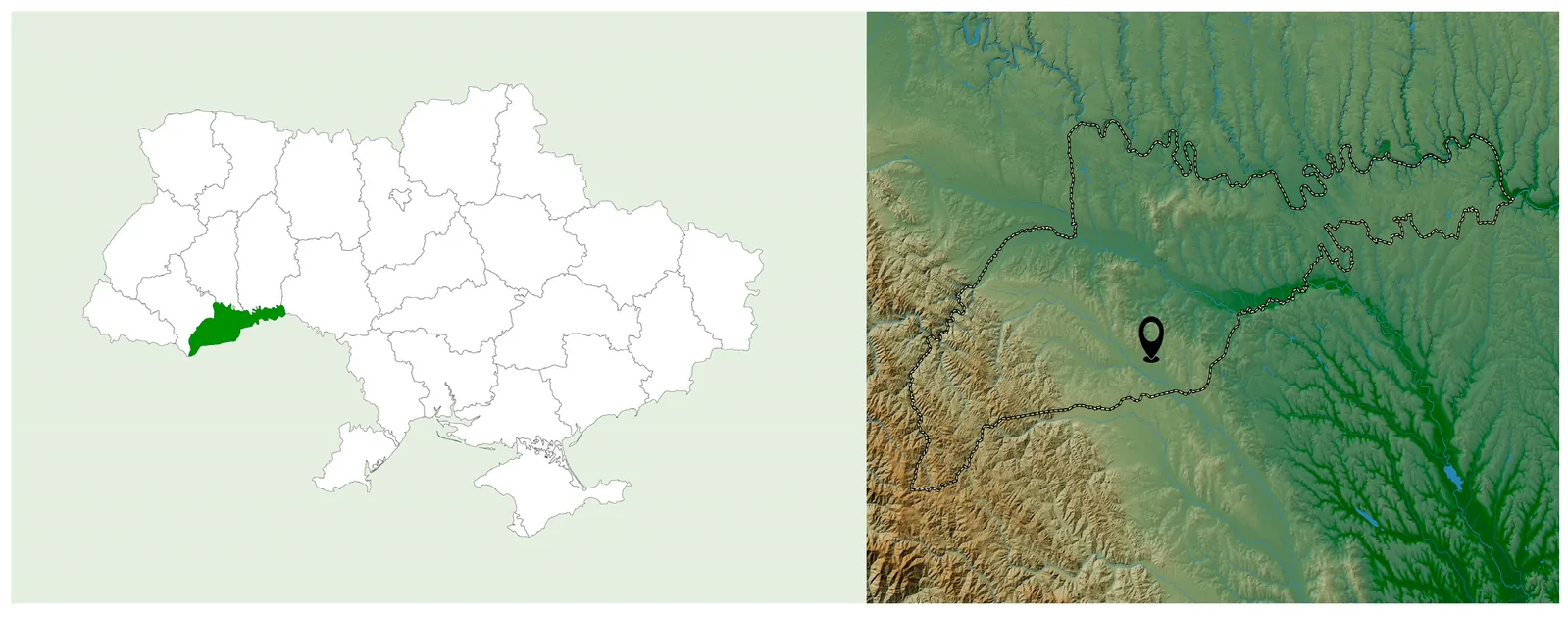
Location of sampling leaves of *Fagus sylvatica* L. in the forest stands of the Bukovinian Pre-Carpathians (Chernivtsi region, Ukraine). The pin indicates the specific research area south of Storozhynets. (Based on a map from Shutterstock, ID 2297931933; sampling sites added by the authors).

Plant material was collected in June–July 2025 in the forest stands of the Bukovinian Pre-Carpathians (Ukraine), within the territory of the State Enterprise “Storozhynets Forestry” (48.077560° N, 25.750491° E). The total area of the forestry enterprise is 35,879.9 ha (Figure 8). Predominant species are European beech (*Fagus sylvatica* L.—39.3%) and silver fir (*Abies alba* Mill.—37.6%). On the selected plots, the average density of beech stands was 0.68. The average height of 100-year-old trees was about 30 m, with trunk diameters of 35–40 cm.

Leaves for morpho-physiological studies were collected from the middle part of the crowns during scheduled tree felling operations. For comparative analysis, trees (up to 80 years old) with wood free of visible defects (control, *n* = 5) and trees exhibiting false heartwood (*n* = 5) were selected. The sampling volume comprised 30 leaves from each tree. After laboratory transportation and subsequent quality screening (excluding samples with mechanical or insect damage), the final analyzed sample size was N = 123 for the HC and N = 131 for the FH morphotype.

### 4.2. Isolation and Identification of Endophytic Microorganisms

The endophytic strain BHFHB was isolated from the wood of *Fagus sylvatica* L. with signs of FH [7,61]. For sequencing, the bacteria were cultivated on potato–glucose agar (PGA) for 48 h. Genomic DNA was extracted from a suspension of bacterial cells using the GeneJet Genomic DNA Purification Kit (Thermo Fisher Scientific Baltics UAB, Vilnius, Lithuania) according to the manufacturer’s protocol. The PCR reaction mixture (25 μL) contained 12.5 μL of 2× DreamTaq PCR Master Mix (ThermoScientific), 30 pmol of each universal primer (27F: 5′-AGAGTTTGATCMTGGCTCAG-3′ and 1492r: 5′-CGGTTACCTTGTTACGACTT-3′), and 50 ng of DNA template. Amplification of 16S rDNA was performed on a Mastercycler Personal 5332 (Eppendorf, Hamburg, Germany). Cycling conditions: initial denaturation—95 °C, 2 min; 30 cycles—95 °C, 30 s; 55 °C, 45 s; 72 °C, 90 s; and final elongation—72 °C, 7 min. The amplicon was separated on a 1.7% agarose gel containing 0.01% ethidium bromide and visualized under UV light. The fragment was excised from the gel and purified using the Zymoclean Gel DNA Recovery Kit (ZymoResearch, Irvine, CA, USA). DNA concentration was measured with a DS-11 FX+ (DeNovix, Wilmington, DE, USA). Sequencing reactions were performed in both directions using the BigDye Terminator v3.1 Cycle Sequencing Kit on a 3130 Genetic Analyzer (Applied Biosystems, Foster City, CA, USA). The complete sequence obtained was compared with deposited sequences using BLASTn (+v. 2.17.0), and phylogenetic analysis was conducted with MEGA X [37]. The data were deposited in GenBank under accession number OR262489.

### 4.3. Anatomical Studies of Plant Tissues

For anatomical investigations, 10 leaves of *Fagus sylvatica* L. were collected. To ensure reproducibility and correct comparison of results, cross sections were made along the central vein using a hand tool, which consisted of two precisely aligned razors, between which an aluminum spacer of 12 μm thickness was placed. The razors were firmly clamped between two plates through technical openings. The distance between the two cutting edges was 13–14 μm. By sliding the device across the central vein, uniform cross-sections of leaf fragments were obtained. Section thickness was controlled with an electronic micrometer (accuracy 0.001 mm). For analysis, sections of 20 ± 3 μm thickness were selected. Anatomical studies and tissue autofluorescence were examined using an inverted microscope equipped with a multichannel fluorescence imaging system (EVOS FL System, Thermo Fisher Scientific, Waltham, MA, USA) with three channels: DAPI (357/44 nm), GFP (482/25 nm), and TexasRed (585/29 nm).

### 4.4. Isolation and Purification of EPS

To obtain EPS, the strain BHFHB was inoculated into 200 mL of LB broth supplemented with 20% sucrose and cultivated (28 °C, 150 rpm, 48 h). Bacterial cells were removed by centrifugation (10,000 *g*, 10 min, 4 °C). EPS was precipitated from the supernatant by adding three volumes of cooled 95% ethanol (24 h, 4 °C). The precipitate was collected by centrifugation (10,000 *g*, 10 min), dissolved in deionized water, and subjected to deproteinization using the Sevag method (chloroform/butanol, 4/1 *v*/*v*). The purified aqueous fraction was re-precipitated with cooled 95% ethanol (24 h, 4 °C); the resulting precipitate was dissolved in water and dialyzed against deionized water (MWCO 3500 Da, Slide-A-Lyzer G2 Dialysis Cassettes, Thermo Fisher Scientific, Waltham, MA, USA) for 3 days at 4 °C. The obtained EPS solution was lyophilized and used for leaf treatments.

### 4.5. Monosaccharide Composition Analysis by GC–MS

A 2 mL aliquot of 2 M trifluoroacetic acid was added to the sample. Hydrolysis was carried out at 100 °C for 6 h. Subsequently, 2 mL of the hydrolysate was collected, evaporated to dryness, and washed with water until complete removal of trifluoroacetic acid. For preparation of aldononitrile acetate derivatives, the extract or hydrolysate was evaporated to dryness using a rotary evaporator, and 0.3 mL of derivatization reagent (32 mg/mL hydroxylamine hydrochloride in pyridine/methanol mixture, 4:1, *v*/*v*) was added. The reaction mixture was incubated for 25 min at 75 °C. Acetylation of aldononitrile derivatives was performed for 15 min at 75 °C. Subsequently, 1 mL of dichloroethane was added, and excess derivatization reagents were removed by double extraction with 1 N hydrochloric acid and water. The dichloroethane layer was evaporated to dryness, and the residue was dissolved in 300 µL of heptane/ethyl acetate mixture (1:1, *v*/*v*).

Chromatographic separation was performed using an Agilent 6890N/5973 Inert GC–MS (Agilent Technologies, Inc., Santa Clara, CA, USA) equipped with an HP-5MS capillary column (30 m × 0.25 mm × 0.25 µm, Agilent Technologies, Inc., Santa Clara, CA, USA). The injector temperature was set at 250 °C and the interface temperature at 280 °C. Separation was carried out under programmed temperature conditions: the initial temperature of 160 °C was maintained for 8 min, then increased at a rate of 5 °C/min to 240 °C, with the final temperature held for 6 min. A 1 µL sample was injected in split mode (1:50). Detection was performed in SCAN mode within the range of 38–400 *m*/*z*. Helium was used as the carrier gas at a flow rate of 1.2 mL/min. Identification of monosaccharides was based on retention times of standard compounds and comparison with the NIST 02 mass spectral library.

### 4.6. Treatment of Leaves with EPS Solution

Freshly collected leaves of *Fagus sylvatica* L. were treated using the application method. Filter paper discs in Petri dishes were soaked with EPS solution (0.1%). Leaves were placed on the paper with the adaxial surface facing down to ensure direct contact of the cuticle with the polysaccharide solution. Incubation was carried out in a thermostat at 25 °C. Control samples were maintained under identical conditions on filter paper soaked with distilled water.

### 4.7. Treatment of Leaves with Ethephon and Sample Preparation

To induce the plant response to ethylene, leaves of model trees *Fagus sylvatica* L.—both control plants and those with signs of FH—were treated in vivo with 10 mM ethephon solution (2-chloroethylphosphonic acid) using a sprayer until the leaf surface was completely moistened. To analyze the flavonoid content dynamics, leaves were sampled immediately before treatment (0 h) and at 1, 2, 3, and 4 h after the treatment. Plant material was dried to constant weight at 60 °C and ground for subsequent extraction.

### 4.8. Sample Preparation and Aqueous–Alcohol Extraction of Compounds

For comprehensive phytochemical analysis of phenolic compounds, leaves of *Fagus sylvatica* L. were dried at 60 °C to constant weight and ground. The resulting dry leaf powder was sieved through a No. 40 mesh. To determine the total phenolic content, 1 g of sample was added to 10 mL (1/10) of methanol/water (80/20, *v*/*v*). To determine the total flavonoid content, the dry leaf mass was extracted with 70% ethanol. Sample mixtures were extracted at 20 °C for 24 h. After extraction, the mixtures were centrifuged at 8000× *g* for 10 min, and the supernatant was collected for analysis. Prior to phytochemical analysis, the samples were stored in a freezer at −20 °C.

### 4.9. Spectrophotometric Determination of Flavonoids and Phenolic Antioxidants

The total flavonoid content in leaves was determined spectrophotometrically using an Optizen Pop spectrophotometer (South Korea). 1.95 mL of deionized water was mixed with 50 μL of ethanolic leaf extract and 75 μL of 5% NaNO_2_ solution. After 5 min of incubation, 75 μL of 10% AlCl_3_ solution was added, followed by 0.5 mL of 1 M NaOH and 0.6 mL of deionized water. Absorbance was measured at 510 nm. A calibration curve (R^2^ = 0.998) was constructed using rutin (0–100 μg ml^−1^).

The concentration of phenolic antioxidants in extracts was determined spectrophotometrically according to the Brand–Williams method using the stable free radical 2,2-diphenyl-1-picrylhydrazyl (DPPH**•**). Trolox ((±) 6-hydroxy-2,5,7,8-tetramethylchroman-2-carboxylic acid, 97%; Aldrich, Milwaukee, Wisconsin) was used as the standard for calibration. The reaction mixture contained 0.25 mL of plant extract, 1.75 mL of 80% ethanol, and 2 mL of 0.2 mM DPPH solution. In control samples, 2 mL of 0.2 mM DPPH solution was mixed with 2 mL of 80% ethanol. Tubes were shaken and left for 30 min in the dark at room temperature. Optical density of the reaction mixture was measured at 515 nm. Antioxidant activity of plant extracts was expressed in μM Trolox equivalents.

### 4.10. Photodocumentation, Digital and Statistical Data Processing

Photodocumentation and digital image processing of leaf blades were carried out using the specialized ImageJ 1.54p software. Statistical processing of the experimental data was performed using the SigmaPlot 12.0 package. The normality of distribution and homogeneity of variances were verified using the Shapiro–Wilk and Equal Variance tests. For macro-morphometric, biochemical, and fluorometric indices, the statistical significance of differences between the studied groups was verified using the non-parametric Mann–Whitney U test or Student’s *t*-test (for indices meeting parametric criteria). Depending on the data presentation format, values are expressed as means ± SD or means ± SE. Differences were considered statistically significant at * *p* < 0.05, ** *p* < 0.01, and *** *p* < 0.001. Multivariate statistical analysis of leaf macro-morphometric indices (PCA, OPLS-DA, and VIP score profiling) was performed using the MetaboAnalyst 6.0 platform.

## 5. Conclusions

Based on histochemical, morphometric, and physiological studies, *Fagus sylvatica* L. trees with signs of FH morphotype exhibit a distinct morphological feature, manifested in a significantly increased LLT and enhanced development of conductive and mechanical tissues in leaves.

The process of FH formation in beech trunks is characterized by pronounced spatial histochemical zonality: in the transition zone (border between dark and light wood), autofluorescent compounds are present in xylem parenchyma and radial rays, whereas in the central part of the trunk’s FH zone, they lose fluorescence capacity due to final polymerization.

The exopolysaccharide of the endophytic strain BHFHB is capable, upon contact with leaf tissues, of initiating processes that strengthen the cell walls of sclerenchyma and xylem within vascular bundles.

Leaves of the FH morphotype trees are characterized by pronounced metabolic hyperactivity in response to ethephon (an ethylene-releasing agent) or the EPS, manifested as a rapid (4–6 h) increase in flavonoid concentration by 20–30% above baseline levels. The similarity in the kinetics of flavonoid accumulation in response to exogenous ethylene and exopolysaccharides suggests a potential common ethylene-dependent mechanism for the perception of MAMPs, which warrants further investigation.

Hypersensitivity to stress-induced ethylene and bacterial elicitors in FH forms of *Fagus sylvatica* L. ensures initiation of heartwood formation significantly earlier than the physiological senescence is reached. The formation of FH in these morphotypes is proposed to be considered as an ecological strategy aimed at optimizing tree viability under extreme conditions of mountain ecosystems; at the same time, the established link between leaf lamina thickness and wood defect predisposition offers a practical, non-destructive proxy for early-stage screening in forest management.

## Figures and Tables

**Table 1:** Morphometric parameters and indices (*n* = 125, m ± SE) describing the ratios and geometry of leaf blades of *Fagus sylvatica* L.

Leaf Sign	HC Morphotype	FH Morphotype	Δ, %
Area, mm^2^	3089.1 ± 132,02	2461.7 ± 365,95	−20.43
Perimeter, mm	231.2 ± 5,91	200.0 ± 15,46	−13.63
Length (L), mm	86.1 ± 1.01	75.2 ± 1.22	−12.82
Width (W), mm	52.5 ± 1.34	46.8 ± 4.26	−11.00
Thickness (LLT), µm	105.8 ± 2,16	126.1 ± 1.45 ***	19.15
Aspect ratio (AR)	1.56 ± 0.015	1.52 ± 0.054	−2.25
Circularity (Circ)	0.72 ± 0.008	0.75 ± 0.008 *	4.43
Roundness (Round)	0.64 ± 0.006	0.66 ± 0.022	2.90
Solidity	0.97 ± 0.002	0.97 ± 0.001	0.14
Dissection index (DI)	1.18 ± 0.008	1.16 ± 0.006 *	−2.12

Note: HC—healthy control morphotype; FH—false heartwood morphotype; Δ, %—relative difference between parameters. Data are presented as means ± standard error(m ± SE). Statistical significance between morphotypes was determined using the Mann–Whitney U test (or Student’s *t*-test where appropriate), with significance levels indicated as * *p* < 0.05 and *** *p* < 0.001.

**Table 2:** Ratios of monosaccharides in the exopolysaccharide of *Bacillus halotolerans* BHFHB.

Monosaccharide	Retention Time (min)	Peak Area	Area (%)
Mannose	15.67	2 524.75	10.36
Glucose	16.07	17 483.56	71.71
Galactose	16.51	1118.54	4.59
Inositol	18.82	3 254.11	13.35

## Data Availability

The original contributions presented in this study are included in the article. Further inquiries can be directed to the corresponding authors.

## Abbreviations

EPS	Exopolysaccharide
FH	False Heartwood Morphotype
HC	Healthy Control Morphotype
LLT	Leaf Lamina Thickness
MAMPs	Microbe Asociated Molecular Patterns
